# VRK3 depletion induces cell cycle arrest and metabolic reprogramming of pontine diffuse midline glioma - H3K27 altered cells

**DOI:** 10.3389/fonc.2023.1229312

**Published:** 2023-10-10

**Authors:** Virginie Menez, Thomas Kergrohen, Tal Shasha, Claudia Silva-Evangelista, Ludivine Le Dret, Lucie Auffret, Chloé Subecz, Manon Lancien, Yassine Ajlil, Irma Segoviano Vilchis, Kévin Beccaria, Thomas Blauwblomme, Estelle Oberlin, Jacques Grill, David Castel, Marie-Anne Debily

**Affiliations:** ^1^ U981, Molecular Predictors and New Targets in Oncology, Team Genomics and Oncogenesis of Pediatric Brain Tumors, INSERM, Gustave Roussy, Université Paris-Saclay, Villejuif, France; ^2^ Department of Pediatric Neurosurgery, Necker Enfants Malades, Paris, France; ^3^ Inserm UMRS-MD 1197, Université Paris-Saclay, Villejuif, France; ^4^ Département de Cancérologie de l’Enfant et de l’Adolescent, Gustave Roussy, Université Paris-Saclay, Villejuif, France; ^5^ Univ Evry, Université Paris-Saclay, Evry, France

**Keywords:** VRK3, diffuse midline glioma H3 K27-altered, oxidative phosphorylation (OXPHOS), cell cycle arrest, pediatric glioma

## Abstract

We previously identified VRK3 as a specific vulnerability in DMG-H3K27M cells in a synthetic lethality screen targeting the whole kinome. The aim of the present study was to elucidate the mechanisms by which VRK3 depletion impact DMG-H3K27M cell fitness. Gene expression studies after *VRK3* knockdown emphasized the inhibition of genes involved in G1/S transition of the cell cycle resulting in growth arrest in G1. Additionally, a massive modulation of genes involved in chromosome segregation was observed, concomitantly with a reduction in the level of phosphorylation of serine 10 and serine 28 of histone H3 supporting the regulation of chromatin condensation during cell division. This last effect could be partly due to a concomitant decrease of the chromatin kinase VRK1 in DMG following *VRK3* knockdown. Furthermore, a metabolic switch specific to VRK3 function was observed towards increased oxidative phosphorylation without change in mitochondria content, that we hypothesized would represent a cell rescue mechanism. This study further explored the vulnerability of DMG-H3K27M cells to VRK3 depletion suggesting potential therapeutic combinations, *e.g.* with the mitochondrial ClpP protease activator ONC201.

## Introduction

Diffuse midline glioma (DMG) H3K27 altered represents the most severe pediatric brain tumor. Almost all the affected children die within 2 years following diagnosis (1, 2) without significant improvement of their overall survival in the last decades despite numerous trials to improve the outcome by adding new agents to the radiotherapy standard (3). These tumors harbor a global loss of K27 trimethylation of histone H3 resulting from the substitution of a lysine residue to a methionine (K27M) in the regulatory tail of histone H3 (4–6) in 90% of cases. This mutation occurs almost exclusively in *H3-3A*, encoding H3.3 histone variant or in *H3C1*, encoding H3.1 variant (4) and is considered the initiating driver event of DMG oncogenesis leading to a broad dysregulation of the epigenome. However, this alteration could not be specifically targeted until now. At present, only secondary driver events are targetable which limits the efficacy of these treatments. Therefore, we performed a synthetic lethality screen by RNA interference targeting the human kinome to unravel genes required for pontine DMG-H3K27M cell survival. We identified *VRK3* (Vaccinia related kinase 3) as an essential gene that has not yet been associated with DMG oncogenesis (7).

VRK3 belongs to the Vaccinia Related Kinase (VRK) family containing 3 distinct members in mammals: VRK1, VRK2 and VRK3 serine/threonine kinases, which are mainly located in the nucleus. VRKs are known to regulate several aspects in cell such as cell cycle progression, nuclear envelop dynamic during mitosis and apoptosis (8, 9). These proteins also play a role in histone post-translational modifications (PTM) (10, 11). In particular VRK1, the most studied member of the VRK protein family, has been shown to bind to chromatin either during mitosis (8) or during DNA damage response (12). VRK1 is in particular required for chromatin compaction in G2/M and during mitosis it progressively regulates histone H3 through its interplay with AURKB, by phosphorylating threonine 3 and serine 10 (Ser10 or S10) residues sequentially. The role of VRK2 has been less investigated, but was associated with a downregulation of apoptosis (13). VRK3 was recently classified as a pseudokinase and remains poorly studied in comparison with other VRK members.

All VRK kinases were shown to phosphorylate BAF a regulator of post-mitotic nuclear envelope formation (14, 15). The partial overlap of VRK1- and VRK3-interacting proteins reflects their common involvement in cell cycle regulation, chromatin assembly and DNA repair (16) while exhibiting distinct roles in these processes. Both VRK1 and VRK3 were shown to promote liver cancer progression, VRK1 regulating G1/S transition and mitosis and VRK3 S phase progression (16). VRK1 was also shown to regulate G1/S transition in fibroblasts (17) but was recently associated with G2/M arrest in glioblastoma (18), suggesting a cell-dependent impact on cell cycle. At a clinical level, *VRK1* overexpression has been associated with poor prognosis in many solid tumors including high-grade glioma (19, 20). We observed that in adult glioma high *VRK3* expression was correlated with decrease in overall survival independently of the grade of the tumor (7), whereas *VRK2* expression was associated with an increase of survival in high-grade astrocytoma (21).

This study aims to improve our knowledge on VRK3 function and the molecular impact of its repression in the context of DMG-H3K27M. We confirmed that *VRK3* repression leads to a strong and rapid arrest of cell growth, resulting in a blockage of the cells in G1 phase and also emphasize a global dysregulation of DMG metabolism.

## Materials and methods

### Cell and culture

Human Glioma Stem Cell primary culture derived in the lab corresponding to three H3.3-K27M (GSC1, GSC2, GSC5) and 2 H3.1-K27M (GSC3, GSC4) cellular models were grown in Neurocult™ medium supplemented with heparin (2 μg/mL, Stemcell Technologies), EGF/FGF (20ng/mL, Miltenyi) at 37°C and 5% CO2 using laminin-coating. Normal stem cell (NSC3, NSC4, NSC5) derived as previously published (7) were grown in the same culture conditions. For GSC cells, PDGF (10 ng/mL, Miltenyi) was added to the culture medium. HEK293T and HCT116 were cultivated in DMEM supplemented with 10% fetal bovine serum and 1% Penicillin/Streptomycin (Life technologies) at 37°C and 5% CO2. For all cell types, medium was renewed in a two-days basis. All the samples were obtained with the written informed consents for the biopsy of subjects according to Helsinki declaration.

### Cell cycle analysis by flow cytometry

Cells were exposed during 2 hours to 10 μmol/L of EdU supplied with Click-iT Flow Cytometry Assay Kit (ThermoScientific) and then detached using accutase^®^ (ThermoFischer Sicentific). EdU was incorporated into cellular DNA during replication to tag cells in S phase. Total DNA was stained with FxCycleTM Violet stain (Thermofisher Scientific). Samples were acquired using an LSR Fortessa flow cytometer (BD Biosciences), and analysis was performed with Flowjow software (Flowjow 10.8.1).

### Lentiviral production and transduction

Lentiviral particles were produced in HEK293T cells using psPax2 and pMD2.g second-generation packaging plasmids (Addgene #12260, #12259) with jetPRIME Polyplus transfection reagent, and lentiviral titers were then determined by fluorescence assay. GSCs were transduced at a multiplicity of infection (M.O.I.) of 3 with shVRK3-1, shVRK3-4, shCtrl-1 (negative control vector containing a nonhairpin insert Addgene #1864) and shCtrl-2 (MISSION^®^ pLKO.1-puro non-mammalian shRNA Control Plasmid DNA, SHC002, Sigma-Aldrich).

### Gene expression of tumor cells after VRK3 silencing with RNA-sequencing

Five hundred thousand cells (GSC1, GSC2, GSC3, GSC4) were transduced (7). Total RNA was extracted with Direct-zol RNA micro prep (Zymo research) at 44 hours and 60 hours post-transduction.

The RNA integrity (RNA Integrity Score≥7.0) was checked on the Agilent 2100 Bioanalyzer and quantity was determined using Qubit (Invitrogen). SureSelect Automated Strand Specific RNA Library Preparation Kit was used according to manufacturer’s instructions with the Bravo Platform. Briefly, 50 to 200ng of total RNA per sample was used for poly-A mRNA selection using oligo(dT) beads and subjected to thermal mRNA fragmentation. The fragmented mRNA samples were subjected to cDNA synthesis and were further converted into double stranded DNA using the reagents supplied in the kit, and the resulting dsDNA was used for library preparation. The final libraries were bar-coded, purified, pooled together in equal concentrations and subjected to paired-end sequencing on Novaseq-6000 sequencer (Illumina). Raw data quality checking was performed with FastQC and pseudo-mapping with Salmon (v0.9.1) using Gencode annotation (v27) based on hg38 reference genome (GRCh38); Genes with less than 10 reads in the resulting raw count matrix were filtered. After variance-stabilizing transformation and removal of sample model bias with limma removeBatchEffect, DESeq2 was used with default parameters to select differentially expressed genes (DEGs) with a pvalue threshold of 0.001 after Benjamini and Hochberg correction. Sample hierarchical clustering was performed using complexHeatmap function with Euclidian distance and complete linkage as aggregation method. For functional annotation and enrichment analysis, we used GeneTonic package, ClusterProfiler package (GO from org.Hs.eg.db GOSOURCEDATA = 2021-02-01) and Gene Set Enrichment Analysis (GSEA) to identify gene sets from the MSigDB v7.4 database (C5 (GO : BP), C2 (KEGG) and HALLMARK catalogue) overrepresented among log2FC ranked genelists, using Benjamini and Hochberg procedure as multiple testing correction. Enrichmap was obtained by computing Jaccard distance between top 100 GOenrich genesets. The communities in Enrichmap were identified by Louvain clustering. Hierarchical clustering of selected gene subsets was conducted using z-score, Pearson correlation and ward D2 as agglomeration criteria.

### DNA methylation array processing

DNA methylation data of the samples of Capper et al. (22) and Castel et al. (23) were analyzed in R V4.0.3. Raw signal intensities were obtained from IDAT files using the minfi Bioconductor package version 1.36.0. Each sample was individually normalized by performing a background correction and a dye-bias correction. Probes located on sex chromosomes, not uniquely mapped to the human reference genome (hg19), containing single nucleotide polymorphisms and that are not present in both EPIC and 450k methylation array were removed. Subsequently, a batch effect correction for the type of material tissue (FFPE/frozen) was performed using limma package v3.30.11). The remaining probes were sorted by standard deviation, the 10,000 most variables were selected and used to compute the 1-variance weighted Pearson correlation between samples. The distance matrix was used as input for t-distributed stochastic neighbor embedding (t-SNE) from Rtsne package, with the following non-default parameters: theta = 0, pca = F, max_iter = 2500 and perplexity = 20. The beta value of the probe cg26093711, localized upstream of the *VRK2* gene, where generated with the function getBeta() from the minfi package.

### Phospho-array

Two-hundreds microliter of lysis buffer are added to cells after *VRK3* silencing and two-and-a-half million cells were scrapped (99003, TPP). Proteins were then extracted and purified using the kit Antibody Array Assay Kit (KAS02) according to manufacturer recommendations (Full moon BioSystem). Fifty μg of proteins were biotinylated, deposited onto the Phospho Explorer Antibody Array containing 1,318 antibodies, hybridized and washed according to manufacturer recommendations. The slides were scanned on a G2505C Agilent scanner at an excitation wavelength of 550nm at a resolution of 10 μm, and images analyzed with Agilent Feature Extraction 12.0 (Agilent Technologies, Inc 2015). The fluorescence signal of each antibody (‘processed signal’) was corrected by subtracting of the local background. Fold change of corrected signal intensity were analyzed in shVRK3-4-transduced cells *versus* corrected signal intensity in shCtrl2 -transduced cells.

### Protein extraction and immunoblotting

For classical protein extraction, cells were lysed as described previously (7) in RIPA lysis buffer. For acidic protein extraction, we used Histone Extraction Kit (Abcam, cat # ab113476) and followed manufacturer protocol. Protein quantification was performed by spectrophotometry with PierceTM BCA Protein Assay Kit (ThermoFischer Scientific, cat # 23225) and proteins were separated on a 4% to 20% polyacrylamide gel (Biorad), and transferred to PVDF membrane (Bio-rad) with a Trans-Blot Turbo system (Bio-Rad). Membranes were incubated overnight at 4°C with primary antibody (VRK3 #HPA056489 Sigma, β-actine #5125 Cell signaling, Lamin # ab16048 Abcam, H3S10P #05-806 Millipore, H4 #07-108 Millipore, H3S28P #07-145 Millipore, VRK1 #HPA000660 Sigma, MSK2 #3679S Cell signaling), and 1 hour at 20°C with horseradish peroxidase-linked secondary antibody for (# 7074S (1/2000), #7076S (1/5000) Cell Signaling Technology), #0500-0099 (1/50000, Biorad). Proteins were enhanced by chemiluminescence reagent (Biorad) and analyzed with ChemiDocTM XRS+ System (Biorad) with Image Lab 4.1 Software (Bio- Rad).

### Immunofluorescence

For immunofluorescence, cells were plated the day before fixation on coverslip coated with 0,1 mg/mL Matrigel (Corning). Fixation was performed for 5 minutes with PBS1X/4%paraformaldehyde, permeabilization for 5 minutes with 0,5% Triton X-100, and blocking for 30 minutes in 1X PBS/5% normal goat serum (ThermoFisher). Cells were incubated overnight at room temperature (RT) with anti-VRK3 (1/1000 #HPA056489, Sigma) and anti-Phalloidine (1/200 #8940S, Cell signaling) and 45 minutes with secondary antibodies (Alexa fluor, Invitrogen 1:800) and Hoechst 33342 (5 ug/mL #H3570, ThermoFisher) before washes. Slides were mounted with fluoromount-G (SouthernBiotech). Images were acquired with Leica SP8 confocal microscope. For immunofluorescence in suspension, cells were plated the day before transduction and fixed 80 hours post-transduction according to ThermoFisher’s protocol. Cells were incubated with antibodies anti-VRK3 (#HPA056489, Sigma), anti-H3S10P (1/1000 #05-806, Millipore), anti-H3S28P (1/1000 #07-145 Millipore) overnight at 4°C and with secondary antibodies (Alexa fluor, Invitrogen 1:800) during 1h at RT. Samples were acquired using an LSR Fortessa flow cytometer (BD Biosciences), and analysis was performed with Flowjow software (Flowjow 10.8.1).

### RT-qPCR

Total RNAs were extracted and purified with Direct-zolTM RNA MicroPrep (Zymo Research) according to manufacturer’s instructions under RNase free conditions. Five hundred ng of total RNA was reverse-transcribed using Revertaid first strand cDNA synthesis kit (Thermo Fisher Scientific). Real time quantification was performed in triplicate with ViiA Real-Time PCR system (ThermoFisher Scientific). Each sample was analyzed in triplicates. Expression of *TBP* (TATA-binding protein) was used as an internal loading control and 2**^-ΔΔ^** CT method was used for relative expression computation. Primer sequences are summarized in **Table S1**.

### Mitotracker staining

Cells were detached with accutase and washed two times with PBS 1X. Cells were then exposed to Green mitotracker™ (Thermofisher, #M7414) during 30 minutes at 37°C and washed two times with PBS 1X. Samples were acquired using an LSR Fortessa flow cytometer (BD Biosciences), and analysis was performed with Flowjow software.

### Mitochondrial function assays

Mitochondrial function assays were performed using MitoPlate S-1(Biolog Inc., Hayward, CA). The substrates on MitoPlate S-1 were first dissolved by incubating the plate with 30 µl of Assay Mix, consisting of 2x BMAS, 6x Redox Dye MC and saponin (30 µg/ml) necessary for cell permeabilization in a 5% CO2 incubator at 37°C for 1 h before inoculating 400,000 cells per well in a volume of 30 µl. Color changes are read kinetically during 24h and are performed with OmniLog instrument (Biolog Inc., Hayward, CA). Signal intensity was measured at 24h and corrected by background subtraction with the negative control condition without substrate. The relative intensity was computed by dividing each intensity by the global mean of a particular sample.

### Drug evaluation

GSC and NSC cells were plated in duplicates at 10.000 - 15.000 cells/cm2 in 96-well plates in 100 μl of complete GSC medium. Twenty-four hours after seeding, Ro31220 (Calbiochem, cat # 557520), SB747651A (Tocris, cat # 4630) or vehicule DMSO (Sigma-Aldrich) were added using the D300E digital dispenser (TECAN). ONC201 (TIC10, Selleckchem cat #S7963) was added with a range of 0.25-8µM. Cell proliferation was assessed during 7 days after treatment by videomicroscopy with an Incucyte Zoom or S3 (Sartorius). Cell confluence was determined by CellPlayer Analysis software (Essen Bioscience) and normalized against the initial time point. Inhibitory Concentration (IC50) values were obtained by plotting logarithmic concentrations of the drug against the area under the curve.

## Results

### Impact of *VRK3* knockdown on DMG, H3K27-altered transcriptome

RNA-seq was performed 44h and 60h post-transduction with two distinct shRNAs targeting *VRK3* in order to evaluate the early impact of *VRK3* knockdown (KD) in four independent *in vitro* models of DMG. *VRK3* expression presented the highest reduction in GSC2 and GSC3 (**Figure S1A**).

**Figure 1:**
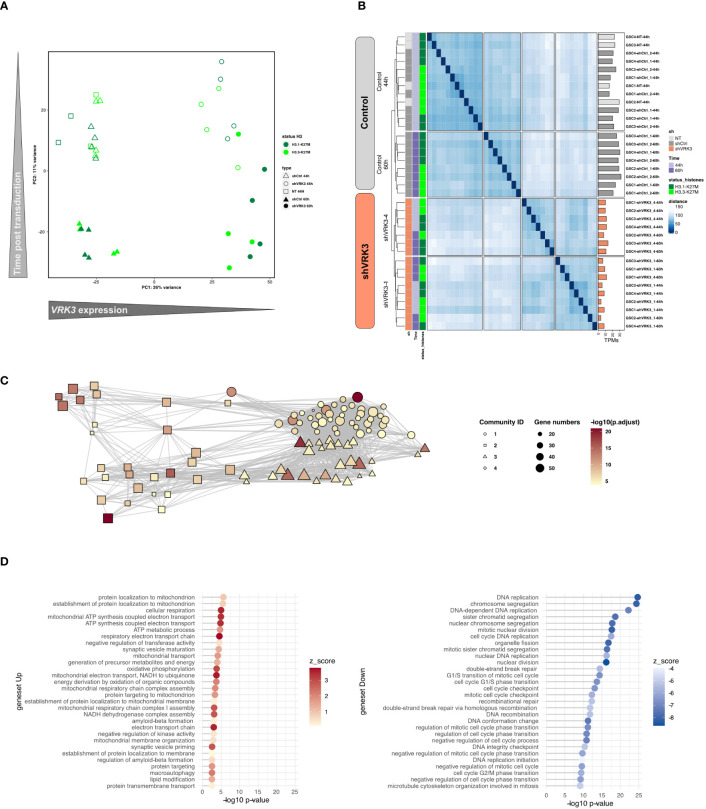
Main pathways affected by *VRK3* repression in DMG-H3K27M. **(A)** PCA analysis of samples based on the entire RNAseq dataset (32 334 genes). **(B)** Sample unsupervised clustering based on the whole transcriptome. The dendrogram illustrates the ordering of patient samples in 2 clusters corresponding to samples transduced with shVRK3 or control samples (not transduced NT or transduced with shCTRL1 or shCTRL2). **(C)** Enrichment map from the top 100 genesets. Relationships between genesets as depicted by Enrich distill analysis. The size of the node encodes the information of the number of DEGs while the color is representative of the computed Z score for each set. The color is indicative of the significance of expression changes (antilog of pvalue) of genes assigned to a particular set. The distinct communities among the enrichment map were identified by Louvain clustering 1) Regulation of cell cycle transition, 2) Telomere organization, 3) Kinetochore and chromatin compaction and 4) Protein localization in mitochondrion. **(D)** Geneset enrichment analysis of DEG associated with adjusted pvalue threshold of 0.001. The 30 most enriched genesets among upregulated (red) and downregulated genes (blue) are presented, showing *e.g.* significance and direction of change (z-score).

The most important source of variation in the data corresponded to the modulation of *VRK3* expression as reflected by PCA analysis based on the whole transcriptome data. Indeed, all samples with *VRK3* KD were well separated from a second group containing both non-transduced cells and cells transduced with non-targeting control shRNAs (**Figure 1A**). Sample unsupervised clustering supported this observation with a clear separation of samples transduced with shVRK3 from the control condition (**Figure 1B**). Two subgroups were observed according to the shRNA targeting *VRK3* used (shVRK3-1 or shVRK3-4), with greater KD with shVRK3-1(**Figure S1A**), even though both targeted the same set of alternative transcripts of *VRK3*.

Samples spread on PC2 depending on the time post-transduction (**Figure 1A**) reflecting the impact of transduction on *in vitro* DMG models. Accordingly, shCTRL samples at 44h or 60h post-transduction distributed in distinct subclusters in consensus clustering (**Figure 1B**). However, differential analysis between shVRK3 cells 44- and 60-hours post-transduction revealed few modulated genes (20 upregulated and 32 downregulated) in comparison with the contrast shVRK3 *versus* shCTRL (1264 upregulated and 1626 downregulated) with the same threshold of adjusted pvalue ≤ 0.001 (**Figures S1B–E**). This suggested that most effect is already present at 44h. Consequently, samples with *VRK3* KD were grouped together for the majority of further differential expression analysis. Functional annotation of differentially expressed genes (DEGs) in shVRK3 cells between the 2 timepoints showed an enrichment of genes involved in histone acetylation with an overexpression of *HDAC5*, *HDAC8*, *TRERF1* and downregulation of *RBM14*; and cholesterol biosynthetic process with a downregulation of *DHCR7*, *NSDHL*, *FASN*, *ACAT2* and *NFYA*. Gene ontology enrichment analyses of DEGs after *VRK3* KD pinpointed a network of the top 100 genesets, from which four communities of highly connected genesets were identified by Louvain clustering: 1) Regulation of cell cycle transition, 2) Telomere organization, 3) Kinetochore and chromatin compaction and 4) Protein localization in mitochondrion (**Figure 1C**). The first three communities present multiple connections with each other’s (**Figure 1C**, **Figure S2**; **Table S2**) while the fourth appears completely independent. Another independent functional annotation identified a larger number of functionally enriched biological processes among repressed genes, in accordance with the volcano plot showing more downregulated DEGs (**Figures S1D, E**; **Table S3**). A massive repression of genes involved in distinct phases of the cell cycle was observed, reflecting that the main impact of *VRK3* KD is cell growth arrest (**Figure 1D**). On the other hand, the upregulated genes emphasized an impact on mitochondrial metabolism.

**Figure 2:**
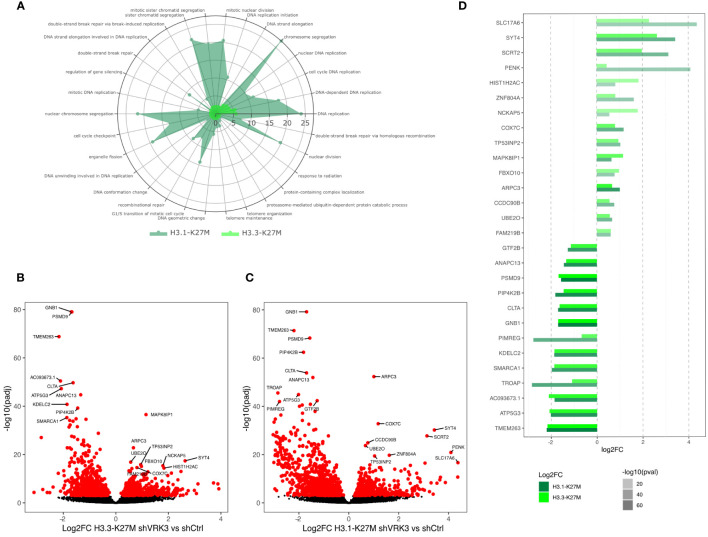
Similar impact of *VRK3* KD in H3.1-K27M and H3.3-K27M cells. **(A)** Radar plot displaying the 30 most enriched genesets in H3.3 GSC following *VRK3* KD ranked by decreasing antilog of pvalue (light green). The corresponding pvalue of overrepresentation analysis in H3.1-mutated tumors are presented (dark green). Functional analysis performed by enrichGO using biological process gene annotation. **(B)** Volcano plot of gene expression in H3.3 shVRK3 cells *versus* H3.3 shCTRL cells plotting antilog of adjusted pvalue on y-axis *versus* log2 Fold Change on x-axis. DEGs associated with adjusted pvalue <0.001 are color-coded in red. The 10 most upregulated and downregulated genes are indicated. **(C)** Volcano plot of gene expression in H3.1 shVRK3 cells *versus* H3.1 shCTRL cells plotting antilog of adjusted pvalue on y-axis *versus* log2 Fold Change on x-axis. DEGs associated with adjusted pvalue <0.001 are color-coded in red. The 10 most upregulated and downregulated genes are indicated. **(D)** Bar charts presenting the expression of genes belonging to the top ten upregulated and downregulated genes in H3.1 (light green) and/or H3.3-mutated cells (dark green) after *VRK3* KD. Gene modulation is presented as the log2FC of RNAseq data on x-axis and the significance is encoded by color transparency.

No major differences between H3.1-K27M and H3.3-K27M cells was observed, as shown by the overlap among the top 20 modulated genes of each subgroup following *VRK3* KD (**Figures 2A–C**). Higher fold changes were present in H3.1-K27M GSC most likely reflecting some heterogeneity between the two H3.3-K27M GSC models (**Figures 2B–D**; **Figure S1C**). In accordance, the same biological processes appeared enriched after *VRK3* repression in the two subgroups even though chromosomal segregation and G1/S transition were impacted to a greater extent in H3.1-K27M cells (**Figure 2A**).

### *VRK3* KD disturbs kinetochore function and chromosome segregation

**Figure 3:**
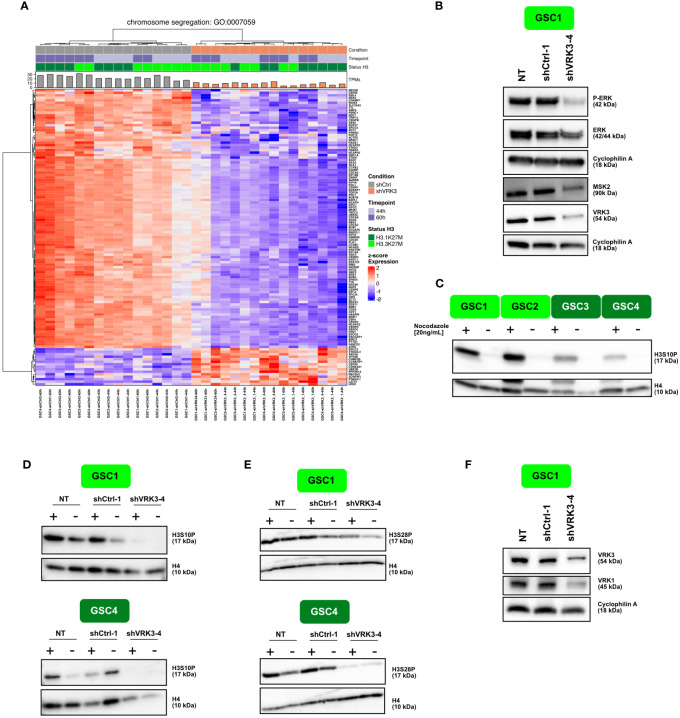
*VRK3* impair chromosome segregation/affect chromatin dynamics in DMG **(A)** Hierarchical clustering and heatmap of modulated genes after *VRK3* KD involved in chromosome segregation (Gene Ontology GO:0044843). **(B)** Protein modulation of VRK3, ERK and MSK2 96 h after transduction with shCTRL-1 or shVRK3-4. Cyclophilin was used as loading control. **(C)** Western Blot analysis showing levels of H3S10P in GSC1, GSC2 (H3.3-K27M model) and GSC3, GSC4 (H3.1-K27M) with or without nocodazole treatment. Histone H4 was used as loading control. **(D, E)** Western Blot analysis showing levels of H3S10P and H3S28P in H3.3-K27M GSC1 and H3.1-K27M GSC4 with or without nocodazole treatment 168h after transduction with shCTRL-1 or shVRK3-4. Histone H4 was used as loading control. **(F)** Protein modulation of VRK1 and VRK3 168h after transduction in GSC1. Cyclophilin was used as loading control.

RNAseq data emphasized a strong impairment of mitosis and more specifically chromosome segregation following *VRK3* KD. Among the top modulated genes, we found a considerable downregulation of clathrin (*CLTA*) and an overexpression of *ARPC3* (**Figure 3A**) involved respectively in the kinetochore stabilization and the dynamics of the actin cytoskeleton during cell division.

VRK3 was previously shown to affect cell proliferation by negatively regulating ERK activity in distinct cell types (10). Therefore, we evaluated the impact of *VRK3* depletion by analyzing the phosphorylation of ERK in H3.3-K27M cells. In our cells, ERK was slightly decreased at protein level, while its phosphorylation was almost fully abolished (**Figure 3B**). ERK inhibition could consequently affect several mitotic kinases operating downstream such as mitogen and stress activated protein kinase MSK1 and MSK2. Among these, MSK2 protein content was decreased in DMG after *VRK3* KD (**Figure 3B**) and a reduction of p-MSK2 was also observed (**Figure S3A**). We showed previously that shRNAs targeting *MSK2* negatively impacted proliferation of DMG cells, but to a lesser degree than *VRK3* KD (7). This is why the impact of pharmacological inhibition of MSK2 using two multi-targeted tyrosine kinase inhibitors was assessed. SB747651A did not present any efficiency in both GSC and NSC cells (IC50 ranging from 15 to 61.3 uM, *data not shown*). For R0318220, NSC4 appeared more sensitive than H3.3- mutated cells which was not concordant with the results of the lethality screen (**Figure S3B**). This discrepancy presumably results from a lack of specificity of the drug or suggests that the strong impact of *VRK3* inhibition in DMG cannot be solely mimicked by MSK2 signaling inhibition.

MSK2 dependent H3S10 phosphorylation (H3S10P) plays a main role in chromatin dynamics and is also tightly associated with cell cycle progression. More precisely, during prophase and metaphase, H3S10P nucleosome occupancy increase strongly along the chromosomal arms (24). Our results showed that H3S10P-positive cells percentage was higher in H3.3-K27M than in H3.1-K27M GSCs and normal NSCs (**Figure S3C**). These differences remained observable after a nocodazole-block in M-phase increasing the global content of H3S10P (**Figure 3C**; **Figure S3D**). Furthermore, *VRK3* KD reduced the pool of H3S10P-positive cells (**Figure 3D**; **Figure S3E**).

MSK2 also induces phosphorylation of both Ser10 and Ser28 of histone H3 when cells are exposed to certain environmental stresses, giving access in regulatory DNA sequences to transcription factors. Similarly to H3S10P, the proportion of H3S28P-positive cells was higher in GSC than in NSC (**Figure S3F**), in particular in H3.3-mutated cells, and decreased following *VRK3* KD (**Figure 3E**; **Figure S3G**). In addition to MSK2, other key mitotic regulators of H3S10P mark deposition were modulated after *VRK3* repression. For example, a strong inhibition of *AURKA* and *AURKB* involved in centrosome maturation and separation, and chromosome condensation respectively was observed. H3S10P is also involved in two structurally opposed processes as it is regulated during transcription activation (25). Indeed, during interphase, H3S10 phosphorylation at certain promoters leads to chromatin remodeling during transcription (24). A previous study of the VRK3 interactome highlighted VRK3 interactions with several chromatin components like histone H1, H2A, H2B type 1 & 2, H3.3, H4 or epigenetic regulators such as Jumonji protein (JARID2), suggesting a role for VRK3 in chromatin organization (16). We found among VRK3 substrates a significant enrichment for genes involved in telomere organization (**Figure S3H**). Also, 24 genes belonging to VRK3 interactome (n=108) were differentially expressed in shVRK3 cells (**Figure S3I**). Among them, *JARID2* encoding a core subunit of PRC2 complex and *HIST1H2AC* encoding histone H2A were upregulated in our study (**Figure 2D**). A significant overlap of VRK3 and VRK1 interactomes was reported (40 common interacting proteins) as well as their direct binding to each other (16). Among the common substrates, four were significantly increased after *VRK3* KD: three ribosomal proteins (RPL11, RPS14 and RPS15A) and NPM1 exhibiting several functions including H3, H4 and H2B core histone chaperoning function. Interestingly, chromatin condensation was reported to be enhanced via phosphorylation of histone H3 on Ser10 by VRK1 (26). In DMG cells, VRK1 harbors mainly a nuclear localization with a significant proportion bound to chromatin (**Figure S3J**), as reported in others cellular contexts (17). In comparison, VRK3 was also mostly found in the nucleus but mainly in the soluble nuclear fraction (**Figure S3K**) although we detected by immunofluorescence a small proportion of VRK3 protein in the cytoplasm, suggesting a potential association with organelles (*data not shown*). Also, VRK2 protein was shown to display a similar cytoplasmic localization, indicating a binding to endoplasmic reticulum and to a lesser extend to mitochondria (27). Strikingly, we observed a decrease of VRK1 protein following *VRK3* KD by RNA interference, even greater than VRK3 itself (**Figure 3F**). The level of expression of both genes was correlated in DMG primary tumors, as opposed to *VRK2* and *VRK3* (**Figure S3L**). Thus, the modulation of H3S10 phosphorylation observed in our cells after *VRK3* KD could result, at least partly, from VRK1 decrease and not only from a direct effect of *VRK3* KD.

### *VRK3* KD impairs DMG proliferation by a massive repression of genes involved in the G1/S cell cycle transition

**Figure 4:**
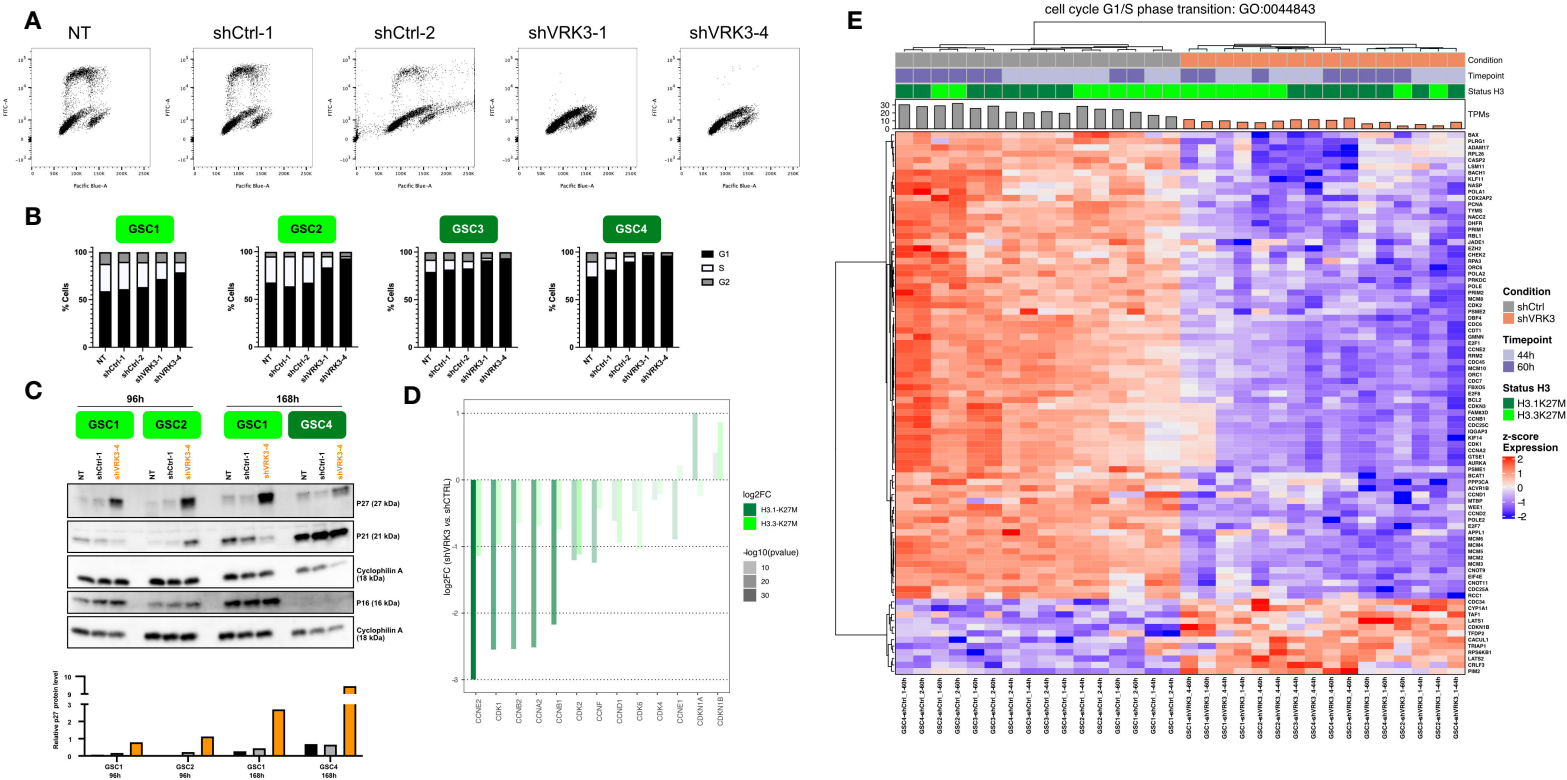
*VRK3* inhibition led to DMG-H3K27M altered G1 growth cell arrest **(A)** Two-dimensional plots (EdU *vs.* FxCycle Violet stain) of GSC4 cells 96h hours after transduction with shCtrl-1, shCtrl-2, shVRK3-1 and shVRK3-4 and quantification of the different cell-cycle population. At least thirty thousand cells were recorded for each condition, triplicates were performed for each GSC model except duplicates for GSC3 cells. **(B)** Histogram presenting percentage of the different cell-cycle populations in GSC cells (H3.3-K27M models GSC1, GSC2 in light green, H3.1-K27M models GSC3, GSC4 in dark green) 96h after transduction at M.O.I. 3 with shCTRL-1, shCTRL-2, shVRK3-1 and shVRK3-4. At least thirty thousand cells were recorded for each condition, quadriplicates were performed for each GSC model except duplicates for GSC3 cells. **(C)** Hierarchical clustering of DEGs in shVRK3 *versus* shCTRL belonging to cell cycle G1/S phase transition geneset (Gene Ontology GO:0007059). Heatmap shows RNAseq expression level (Z-score on normalized expression matrix) **(D)** Bar charts presenting modulation of CDK and CDK inhibitors in H3.3 and H3.1-mutated cells after *VRK3* KD. Gene modulation is presented as the log2FC of RNAseq data on y-axis and the significance is encoded by color transparency. **(E)** Western Blot analysis showing levels of P27, P21 and P16 in GSC1 and GSC2 (H3.3-K27M model) and GSC4 (H3.1-K27M) 96h or 168h after transduction with shCtrl-1 or shVRK3-4. Cyclophilin was used as loading control. Densimetric quantification of western blot was processed with Image Lab and P27 protein level was normalized to cyclophilin expression.

Overall, *VRK3* inhibition led to G1 growth arrest as reflected by the significant increase of the percentage of G1 cells and loss of cells in S phase 96h after transduction (**Figures 4A, B)**. We observed some variations between GSC models in the distribution of cells among the distinct phases of cell cycle even without shVRK3 transduction. GSC1 appeared less sensitive than the others to shVRK3, possibly linked to a lower *VRK3* KD in these cells (**Figures 4A, B**; **Figure S1B**). In non-transduced cells, we did not notice much variations in *VRK3* expression level during the cell cycle. Yet, a substantial increase was measured in G2 phase both at transcriptional (**Figure S4A**) and protein level (**Figure S4B**).

In accordance with cell cycle modifications, RNAseq data showed a significant modulation of genes involved in G1/S transition (**Figures 1D**, **4C**) as early as 44h after transduction and to a greater extent in H3.1-mutated cells (**Figures 2A**, **4D**). All cyclins and CDKs were downregulated, with a huge repression of cyclin E2 (*CCNE2*) and *CDK2* in both H3.1 and H3.3 cells. Conversely, *CDKN1B* encoding P27 was upregulated (**Figure 4D**). Accumulation of P27 was confirmed by western blot analysis and appeared proportional to the percentage of cells in G1 (**Figure 4E**). P27 is known to prevent the G1/S transition by repressing the CDK4-Cyclin D1 (*CCND1*) and CDK2-cyclin E1 (*CCNE1*) protein complexes. S phase entry is mainly under the control of cyclin D/CDK4/6 activation in early G1 leading to activation of CDK2/Cyclin E in late G1 (28). Accordingly, we also observed a downregulation of *CCND1* in all DMGs preventing S phase entry (**Figure 4D**). P27 controls G1 phase along with ERK protein kinase and, as mentioned previously, we measured a significant decrease of p-ERK in shVRK3 cells (**Figure 3B**). The modulation of the CDK inhibitor *CDKN1A*, which encodes P21 involved in G1 block through the inhibition of CDK2-cyclin E1 complexes, was more heterogenous between GSC cells. Indeed, *CDKN1A* and *CCNE1* showed an opposite behavior at RNA level following *VRK3* KD: *CDKN1A* was upregulated in H3.1-mutated cells and slightly downregulated in H3.3-mutated cells; *CCNE1* was downregulated in H3.1- and slightly upregulated in H3.3-mutated cells (**Figure 4D**). WB analysis confirmed the induction of P21 in H3.1-mutated GSC4, and its reduction in H3.3-mutated GSC1. However, it also showed a small increase in P21 content in GSC2 (**Figure 4E**). The decrease in P21 protein content observed specifically in GSC1 cells could result from a milder knockdown of *VRK3* in these cells and a higher proportion of cells remaining in S phase (**Figure 4B**). While a direct binding to CCNB1 and its phosphorylation by VRK3 was previously reported (16), we observed here a decrease of *CCNB1* in DMG cells after *VRK3* KD. CDKs are regulated by several kinases in particular *WEE1*, likewise decreased in shVRK3 cells. Additional proteins linked to progression through G1 phase of the cell cycle are greatly modulated following *VRK3* KD (**Table S3**). Among them, we can mention the strong downregulation of *ANAPC13*, a component of the Anaphase Promoting Complex that additionally controls progression through mitosis (**Figure 2D**).

### Gene overexpression after *VRK3* KD mainly restricted to metabolism associated genes

**Figure 5:**
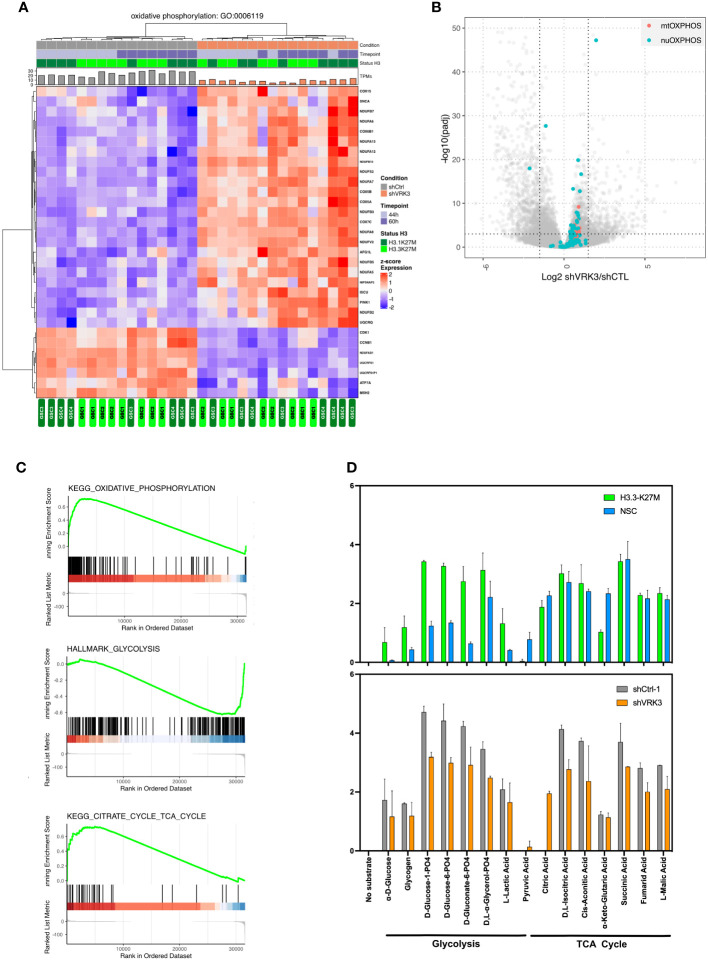
Impact of *VRK3* on metabolism in DMG-H3K27M **(A)** Hierarchical clustering and heatmap of modulated genes after *VRK3* KD involved in oxidative phosphorylation (Gene Ontology GO:0006119). **(B)** GSEA plots showing KEGG oxidative phosphorylation, citrate cycle and Hallmark glycolysis pathways in GSC cells following *VRK3* KD. **(C)** Modulation of both nuclear and mitochondrial genes related to OXPHOS. Volcano plot of gene expression in shVRK3 *versus* shCTRL cells plotting antilog of adjusted pvalue on y-axis *versus* log2 Fold Change on x-axis. Nuclear (nuOXPHOS) and mitochondrial (mtOXPHOS) genes are depicted in blue and red respectively. **(D)**The rate of oxidation of a panel of different types of substrates was assessed using Biolog Mitoplate S1 assay. The relative intensity of each substrate is shown as histogram, error bar reflecting the standard deviation of replicate experiments, *i.e.* H3.3-K27M cells (n=2) and NSC cells (n=2), (GSC n=2 for each condition NT, shCTRL1, shVRK3).

As mentioned previously, RNAseq data suggest metabolic reprogramming in cells following *VRK3* repression, as reflected by a significant overexpression of genes involved in ATP-synthesis coupled electron transport and oxidative phosphorylation (OXPHOS) (**Figures 1D**; **5A, B**). Additionally, *ATP5G3* a metabolism-associated gene is among the top 10 modulated genes (**Figure S1D**; **Figure 2D**). Our results also showed a global increase of mitochondrial RNA content in shVRK3 *versus* shCTRL cells (**Figure S5A**). Consequently, we wondered if the overexpression of OXPHOS-related genes only resulted from transcriptional modulations or could also reflect a variation in term of mitochondrial DNA (mtDNA) content. Indeed, such large variations in mitochondria copy number were reported in some tumors (29). Additionally, Shen and *coll.* recently reported a decrease of mtDNA copy number in DIPG post-mortem samples *versus* normal brain, but using autopsy samples of frontal lobes consisting primarily of gray matter as reference samples (30). This is why the mtDNA copy number in DMG-H3K27M altered primary tumors (n=15) compared to non-tumoral pontine tissue samples (n=6) was evaluated. Despite variability among patients, we measured a tendency for decreased mtDNA content in DIPG either by QPCR (**Figure S5B**) and WES analyses (*data not shown*), confirming the observation of Shen and *coll*. This observation is in accordance with a higher mitochondrial RNA content in non-tumoral pontine tissue samples in comparison with DMG-H3K27M primary tumors (**Figure S5C**). However, no modulation of mtDNA content that could explain upregulation of OXPHOS genes after *VRK3* repression was identified by QPCR even 120h after transduction (*data not shown*). Around 10% of OXPHOS genes (13/132 in KEGG oxidative phosphorylation pathway) are encoded by the mitochondrial genome. Looking independently at the modulation of the two subsets of OXPHOS related genes, an upregulation restricted to mtOXPHOS genes in DMG *versus* normal NSC was identified (**Figure S5D**). In contrast, both subsets were significantly upregulated in GSC after *VRK3* depletion (**Figure 5C**) suggesting a specific and global impact of *VRK3* depletion in the induction of this pathway. Among these DEGs, *COX7C* encoding the Cytochrome C oxidase subunits VIIC of the electron transfer respiratory chain (ETC) was one of the most significantly upregulated genes in shVRK3 cells (**Figure 2D**). We investigated if the deregulation of oxidative phosphorylation following *VRK3* repression was more extensively linked to modifications of cellular metabolism by GSEA analysis of RNAseq data. The results confirmed the enrichment in shVRK3 cells of the ‘oxidative phosphorylation’ geneset, highlighted the enrichment of ‘TCA cycle related genes’ and a tendency for a depletion of genes involved in ‘glycolysis’ (**Figure 5B**; **Figure S5E**). The modulation of individual selected genes in metabolic pathways showed a significant upregulation of majority of genes in OXPHOS, *GALT* in galactose metabolism and *SLC2A3* and *PGAM2* in glycolysis (**Figure S5E**). Other glycolysis-related genes harbored opposite modulation, *i.e. PGK1*, *PFKFB3* and *PFKFB4*. Of note, PFKFB4 is a regulatory enzyme that synthesizes a potent stimulator of glycolysis. Its repression was shown to suppress tumor growth and metastasis in breast cancer (31), and is considered as a key molecule to impair glioma proliferation (32) and survival of cancer stem cells in glioblastoma (33). Indeed, PFKFB4 enhances G1/S transition by increasing CDK6 level (34).

We next investigated mitochondrial metabolic activity and substrate preference including TCA cycle intermediates, hexoses, trioses, amino acids and fatty acids. The rate of substrate oxidation of amino acid and fatty acid substrates were close to background level, and appeared significantly decreased for glycolysis and TCA cycle substrates after *VRK3* KD (**Figure 5D**). These results confirmed a lower glycolysis in GSC after *VRK3* KD, as suggested by GSEA analysis. Interestingly, we observed a global increase of glycolysis in GSC *versus* NSC, *VRK3* repression tending to reset this metabolic pathway. The decrease in the rate of oxidation of several substrates of the TCA cycle, such as fumaric and isocitric acids suggests a reduction of this metabolic activity despite an enrichment of several upregulated genes in shVRK3 cells highlighted by GSEA.

The imipridone ONC201, a potent activator of the mitochondrial Clp protease proteolytic subunit (ClpP), was shown to inhibit cell proliferation and induce cell death in many cancers including GBM (35) and one DMG patient (36). ONC201 lead to a decrease expression of respiratory chain proteins and subsequently impair OXPHOS that results in mitochondrial dysfunction and tumor cell death. We tested ONC201 in our GSC models (n=10, 5 H3.1- and 5 H3.3-mutated cells) and found IC50 ranging from 0.95 to 1.7 µM (median 1.35 µM, *data not shown*). This significant efficiency appeared independent of *EGFR* expression level in the corresponding primary tumors despite the fact that its efficiency was reported to be inversely proportional to *EGFR* expression in GBM (37).

## Discussion

Our gene expression study gave some clues to explain the profound impact of VRK3 deprivation on DMG cell fitness. There were only few differences between the two main subgroups of DMG-H3K27 altered, *i.e.* H3.1- and H3.3-K27M DMG, despite that the H3.3 mutated GSC models used were *TP53*-mutated and that *VRK3* was shown to interact with TP53 protein. A massive gene downregulation affected genes involved in chromatin segregation.

Chromatin condensation, one of a crucial step in mitosis, is known to be enhanced via phosphorylation of histone H3 on Ser10. This modification is critical during neoplastic transformation, and the steady state level of H3S10P is elevated in oncogene-transformed cells and human tumor cell lines (38, 39). Accordingly, we observed a higher content of H3S10-positive cells in H3.3-K27M GSC *versus* NSC. *VRK3* inhibition in DMG led to a significant decrease of H3S10P-positive cells. However VRK3 protein appeared unbound to chromatin in DMG-K27M in contrast to VRK1, as also reported in fibroblasts (17). Consequently, we can suppose that VRK3 is not directly involved in the reduction of H3S10 phosphorylation, and that it could at least partially result from a decrease of *VRK1*. Indeed, VRK1 progressively regulate histone H3 during chromatin compaction in G2/M and mitosis (40). In mammals, H3 phosphorylation occurs at two serine residues, S10 and S28, which can be mediated by histone kinases including MSK1 and AURKB. In shVRK3 cells, both modifications were decreased in parallel with modulation of several of key regulators of H3S10 phosphorylation (AURKA, B, MSK2, 14-3-3).

H3S10P is also involved in chromatin dynamics and was reported to prevent deposition of H3K9me2 (24). VRK3 is known to interact directly with several histone proteins and chromatin modifiers (16). Here, we show modulation of genes involved in histone acetylation 60h after *VRK3* KD, an inhibition of *JARID2* encoding a component of PRC2 complex, and a strong decrease of *SMARCA1* encoding an ATPase of the ISWI subfamily of chromatin remodelers SWI/SNF. All these observations support its role in chromatin dynamics beyond the modulation of H3S10P. Yet, an interactome analysis in DMG cells would be needed to allow the identification of the direct targets of VRK3.

Many genes linked to cell proliferation were also downregulated in DMG cells, explaining the major phenotypical impact of *VRK3* depletion as a G1 block. Another study in liver cancer reported a growth inhibition following *VRK3* silencing (16) but resulting from arrests in S and G2/M phases. This discrepancy could reflect a substantially distinct role of VRK3 in different cellular contexts. These authors have also shown that VRK1 plays a crucial role in G1/S transition and that its depletion induces a G1 arrest by downregulating cyclin D1 and phospho-RB, and upregulating P21 and P27 in hepatocellular carcinoma (16). In DMG, the substantial decrease of VRK1 upon *VRK3* KD is associated with the upregulation of P21 and P27 and downregulation of *CCND1.* One can hypothesize that the observed cell cycle blockade occurs through VRK1 downregulation.

Recently VRK1 was shown as a synthetic lethal target in VRK2-deficient glioblastoma (18) and more globally in VRK2-promoter methylated cancers of the central nervous system (41), including DMG. In the second study, they showed that VRK1 dependency was inversely correlated with expression of *VRK2*, and that *VRK2* promoter methylation was associated with low *VRK2* expression level mainly in G34R/V diffuse hemispheric gliomas. In contrast with *VRK3*, *VRK1* KO led to cell death without any alteration of cell cycle profile in DMG models. In our models we did not notice an hypermethylation of *VRK2* promoter (**Figure S3M**) and observed a higher correlation between *VRK3* and *VRK1* expression in comparison to *VRK3* and *VRK2*. Our previous work has showed that our DMG-H3K27M models are more dependent on *VRK3* than *VRK1* or *VRK2* (7). Indeed, we also observed that *VRK1* extinction by RNA interference affect DMG survival but to a significant lesser extent than VRK3. This observation reflects that it is unlikely that VRK1 can fully compensate VRK3 function, making of VRK3 a more interesting target in DMG-H3K27M. It would thus be interesting to investigate if *VRK3* depletion is also synthetic lethal in other pediatric tumor entities without hypermethylation of *VRK2* promoter, and in particular posterior fossa type A ependymoma and infant high-grade glioma (**Figure S3M**). In this context, it will also be pivotal to confirm the ability of VRK3 targeting to lead to tumor regression *in vivo* through the use of patient-derived xenograft models.

VRK1 and VRK3 share some common substrates (16), but beyond their overlapping role on cell cycle progression, VRK3 presents other specific function impairing DMG-H3K27M survival. Interestingly, previous comparison of VRK1 and VRK3 interactomes uncovered that VRK3 interacts specifically with proteins located in endoplasmic reticulum and mitochondria (16).

Metabolic reprogramming is a hallmark of cancer cells, that results in many tumors from a variation in mtDNA copy number content. A decrease of mtDNA copy number is observed in the majority of tumors (29). Similarly, DMG harbor a lower mtDNA copy number than control NSC cells which is not altered by *VRK3* repression. Most tumors, including adult glioblastoma (aGB), show important indicators of reduced OXPHOS system activity compared with that of the electron transport system. Indeed, glioma cells predominantly rely on glycolysis instead of oxidative phosphorylation to regenerate ATP (42–44). This phenomenon, named the Warburg effect (45), results from the abnormal availability of respiratory substrates. Accordingly, we observed in DMG-H3K27M cells a disruption of the energy metabolism favoring glycolysis over OXPHOS in comparison to NSC control cells. We partially validated the results of Chung and coll., generated by comparing isogenic NSC cells transduced or not with H3K27M construct, with only an increase of glycolysis in K27M cells (46). Knocking-down *VRK3* tends to revert this metabolic phenotype and leads to growth arrest. *VRK3* KD affects metabolism in particular through an upregulation of the majority of respiratory electron transport chain genes as well as OXPHOS related genes. Shen and *coll.* have shown that PDK inhibitors are also able to revert the Warburg effect by stimulating OXPHOS in DMG and restore mitochondrial metabolism, which was associated with a decrease cell viability (30). HDAC inhibitors revert the Warburg effect through a reduction in glycolysis leading to energy deprivation, which in turn leads to enhancement of oxidative metabolism (47). Additionally, it was recently shown in aGB that the activation of ClpP through utilization of the second-generation compounds (ONC206 and ONC212) in combination with pharmacological inhibition of HDAC1/2 cause synergic reduction of viability (48). Lately, ONC201, a potent activator of the mitochondrial ClpP leading to OXPHOS impairment and decrease in enzymatic activity of respiratory chain complexes I, II, and IV, was proposed as an anticancer strategy in DMG-H3K27M (49). Accordingly, ONC201 appeared efficient *in vitro* in all DMG-H3K27M GSC models tested. This family of compound was however less efficient in aGB unless OXPHOS was upregulated (48), and ONC201 has been shown to be more effective in AML which were dependent to OXPHOS (50). Our data support that DMG-H3K27M present a dependency on OXPHOS as well and that strong up-regulation of OXPHOS genes after *VRK3* KD is associated with impairment of their growth. It could thus be anticipated that combining ONC201 with pharmacological targeting of VRK3 would be synergistic.

In summary, VRK3 has non-redundant functions with VRK1 and VRK2 specifically on metabolism. This needs to be further explored through in-depth evaluation of mitochondrial respiration capacity of DMG-H3K27M following VRK3 depletion. Our data clearly reinforce the interest of VRK proteins as therapeutic targets in cancers from nervous system and in particular of VRK3 in DMG and other tumor entities not associated with VRK2 repression by DNA methylation.

## Data availability statement

The datasets presented in this study can be found in online repositories. The names of the repository/repositories and accession number(s) can be found below: https://ega-archive.org, EGAS00001007047.

## Ethics statement

The studies involving humans were approved by informed consent for the Translational Research Program was obtained from parent or guardian according to the IRB approved protocol (CNIL 1176643). The studies were conducted in accordance with the local legislation and institutional requirements. The human samples used in this study were acquired from primarily isolated as part of our previous study for which ethical approval was obtained.

## Author contributions

VM, DC, JG, and M-AD contributed to conception and design of the study. VM, TS, LA, CS, ML performed experiments. TK and YA performed the bioinformatic analysis. VM and M-AD wrote the manuscript. All authors contributed to the article and approved the submitted version.
