# Supplementary material for: VRK3 depletion induces cell cycle arrest and metabolic reprogramming of pontine diffuse midline glioma - H3K27 altered cells
Source: Front Oncol. 2023 Oct 10;13:1229312. doi: 10.3389/fonc.2023.1229312 (PMC10599138; doi:10.3389/fonc.2023.1229312)
Supplement: Supplementary file 1 [file DataSheet_1.zip › Supplementary Figures/Supplementary Figures Caption.docx]

**Supplementary Figure Caption**

**Figure S1**

A-*VRK3* expression level in RNAseq (tpm) is significantly decrease in shVRK3 samples (orange) in comparison to shCTRL samples (dark gray, Mann-Whitney, pvalue 0.0159) both for H3.1- (light green) and/or H3.3-mutated cells (dark green). NT, non-transduced cells in light grey

B. Distribution of Differentially Expressed Genes (DEGs) following *VRK3* KD selected with an adjusted pvalue threshold of 0.001 according to the time post-transduction.

C. Distribution of DEG following *VRK3* KD selected with an adjusted pvalue threshold of 0.001 according to H3.1 and H3.3 mutational status (*i.e.* dark green for DEGs found specifically in H3.1-K27M cells, light green for DEGs found specifically in H3.3-K27M cells and red for common DEGs).

D. Volcano plot of gene expression in shVRK3 cells 60h *versus* shVRK3 cells 44h post-transduction plotting antilog of adjusted pvalue on y-axis *versus* log2 Fold Change (FC) on x-axis.

DEGs associated with adjusted pvalue <0.001 are color-coded in red.

E. Volcano plot of gene expression in shVRK3 cells *versus* shCTRL cells, plotting antilog of adjusted pvalue on y-axis versus log2 Fold Change on x-axis. DEGs associated with adjusted pvalue <0.001 are color-coded in red.

**Figure S2**

Representation of the four distinct communities identified by Louvain clustering among the enrichment map presented in figure 2.

The size of the node encodes the information of the number of DEGs while the color is representative of the computed Z score for each set. The color is indicative of the significance of expression changes of genes assigned to a particular set.

**Figure S3**

A- Modulation of phospho-MSK2 level depicted by phospho-array analysis 72h after transduction with shCTRL-2 or shVRK3-4 in H3.3-K27M GSC2 (ligh green) and H3.1-K27M (dark green) GSC4 cells. Error bars represent standard deviation of experimental duplicates.

B- Dose-response results displaying the effect of MSK2 inhibitor on NSC and GSC proliferation. Proliferation was monitored by video microscopy for 168 hours. Normalized areas under the curve (AUC) were plotted against logarithmic concentrations of Ro318220 drugs to determine the IC50. H3.3-K27M GSCs are shown in light green and H3.1-K27M GSC in dark green.

C- Mean of percentage of H3S10P-positive cells assessed by immunofluorescence in several experimental replicates (*i.e.* GSC1/2/4 n=5, GSC3 n=2 and for control samples NSC4/5 n=4,). Error bars correspond to standard deviation of experimental replicates. * t-test pvalue<0.05, ** t-test pvalue<0.005.

D- Western Blot analysis showing levels of H3S10P in GSC1, GSC2 and GSC5 (H3.3-K27 model, light green) and NSC3 with or without nocodazole treatment. Histone H4 was used as loading control.

E- Modulation of the percentage of H3S10P-positive cells after *VRK3* KD in several experimental replicates (*i.e.* GSC1 n=3, GSC2/3 n=1, GSC4 n=2). * t-test pvalue<0.05, ** t-test pvalue<0.005, *** t-test pvalue<0.0005.

F- Mean of percentage of H3S28P-positive cells assessed by immunofluorescence in several experimental replicates (*i.e.* GSC1/2/4 n=4, GSC3 n=1 and for control samples NSC4/5 n=3). Error bars correspond to standard deviation of experimental replicates. * t-test pvalue<0.05, ** t-test pvalue<0.005,

G-Modulation of the percentage of H3S28P-positive cells after *VRK3* KD in several experimental replicates (*i.e.* GSC1 n=3, GSC2/3 n=1, GSC4 n=2). * t-test pvalue<0.05, ** t-test pvalue<0.005, *** t-test pvalue<0.0005.

H- Analysis of biological process enrichment of genes encoding VRK3 interacting proteins from Lee and coll. ^16^. Using enrichGO function of cluster profiler package. Adjusted pvalue from Fisher exact test are color-coded.

I- Hierarchical clustering of gene encoding VRK3 interacting proteins identified by Lee and coll. ^16^. Heatmap show RNAseq expression level (Z-score on normalize expression matrix) and on the left of the heatmap differentially expressed genes identified using either an adjusted pvalue threshold of 0.01 or 0.001 are indicated as red squares.

J-K- Analysis of the subcellular location of VRK1 and VRK3 by Western Blot in total, cytosolic, nuclear soluble and nuclear insoluble fractions in GSC2 and GSC4 cells. β–Actin, lamin B1 and H4 were used as loading control for total/cytosol, nuclear soluble and nuclear insoluble fractions respectively.

L*-* Dot plot presenting *VRK1* *vs.* *VRK3* expression levels on the upper panel and *VRK2* *vs. VRK3* expression levels on the lower panel from RNAseq data of primary DMG (tpm). Spearman (R) and Pearson correlation (p) are indicated.

M- t-SNE analysis of DNA methylation profiles of 974 gliomas selected reference samples. Left panel shows the distribution of the samples colored by their methylation beta value for the probe cg26093711 located in *VRK2* promoter region. Right panel shows the same samples colored by tumor entity.

**Figure S4**

Modulation of *VRK3* expression during cell cycle assessed by qRT-PCR (A) or immunofluorescence (B). The mean fluorescence intensity of cells across cell cycle was corrected by fluorescence intensity in secondary antibody alone. One-way ANOVA was performed only for GSC cells with at least two independent experiments. One-way ANOVA ** pvalue < 0,01 **** pvalue < 0,0001. Light green, H3.3-K27M GSCs and dark green H3.1-K27M GSC.

**Figure S5**

A- Boxplot reflecting global level of expression of mitochondrial RNA in GSC transduced with shCTRL or shVRK3 (tpm from RNAseq data) (Wilcoxon test *pvalue<0.05, **<0.005).

B- Relative mtDNA copy number measured by qPCR in primary tumors (pontine DMG-H3K27M altered n=15) and non-tumoral primary pontine tissue samples (NC ou NonContrib, n=6) of DMG-H3K27M patients. Error bars represent standard deviation between samples.

C- Violin plot of global level of expression of nuclear and mitochondrial transcriptomes in DMG-H3K27M primary tumors (n=15) and non-tumoral primary pontine tissue samples (NonContrib, n=3) (tpm from RNAseq data). Error bars represent standard deviation between samples.

D- Volcano plot of nuclear and mitochondrial genes related to OXPHOS in GSC cells *versus* NSC cells. Nuclear (nuOXPHOS) and mitochondrial (mtOXPHOS) genes are depicted in blue and red respectively.

E- Barplots presenting the fold change of metabolism genes between normal and glioma stem cells (green) as well as in GSC after *VRK3* KD (orange). Benjamini and Hochberg adjusted pvalue * < 0.05, ** < 0.01, *** < 0.001.
