# Supplementary figures and images for: VRK3 depletion induces cell cycle arrest and metabolic reprogramming of pontine diffuse midline glioma - H3K27 altered cells
Source: Front Oncol. 2023 Oct 10;13:1229312. doi: 10.3389/fonc.2023.1229312 (PMC10599138; doi:10.3389/fonc.2023.1229312)

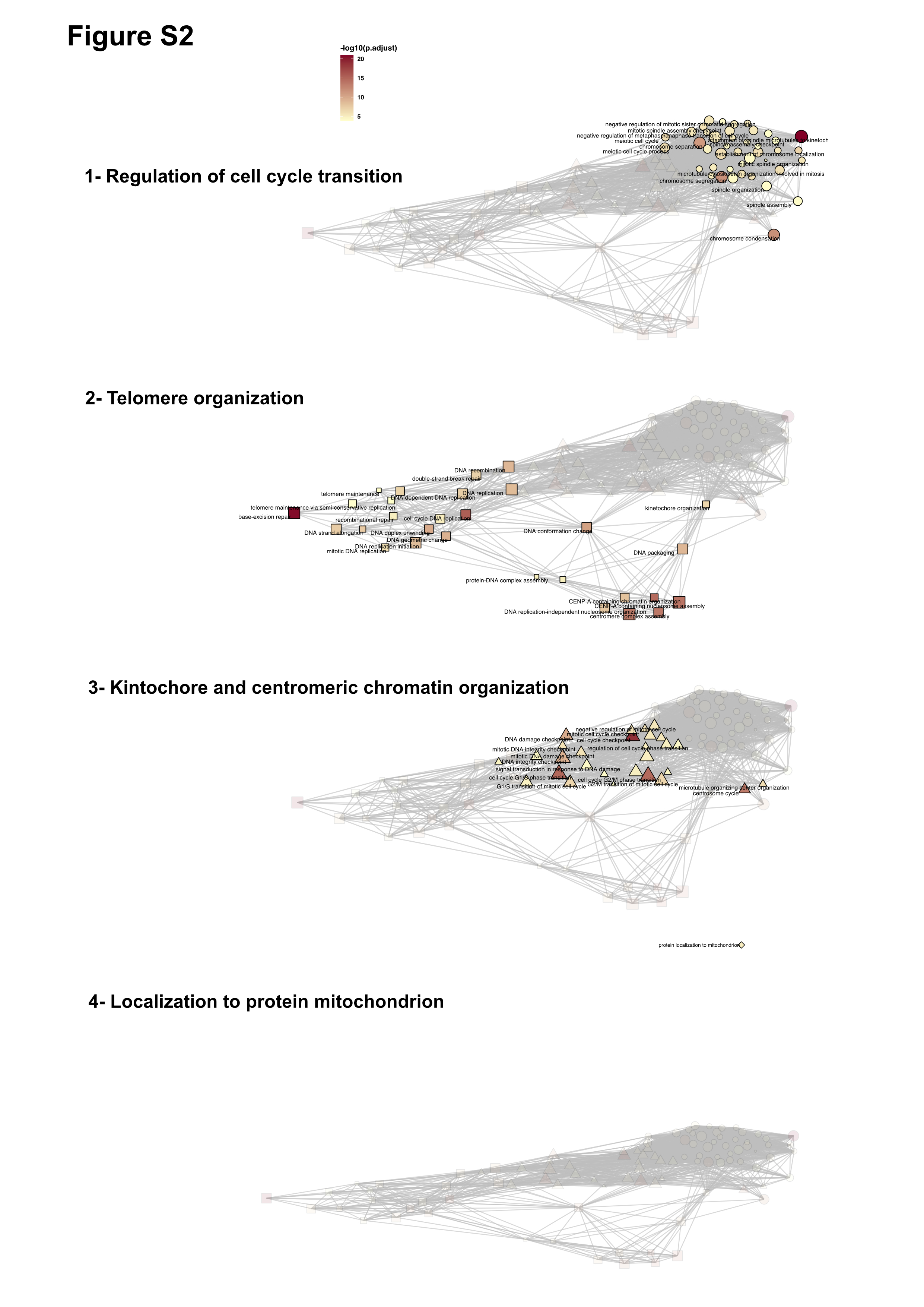

Supplement: Supplementary file 1 [file DataSheet_1.zip › Supplementary Figures/Image 2.JPEG]

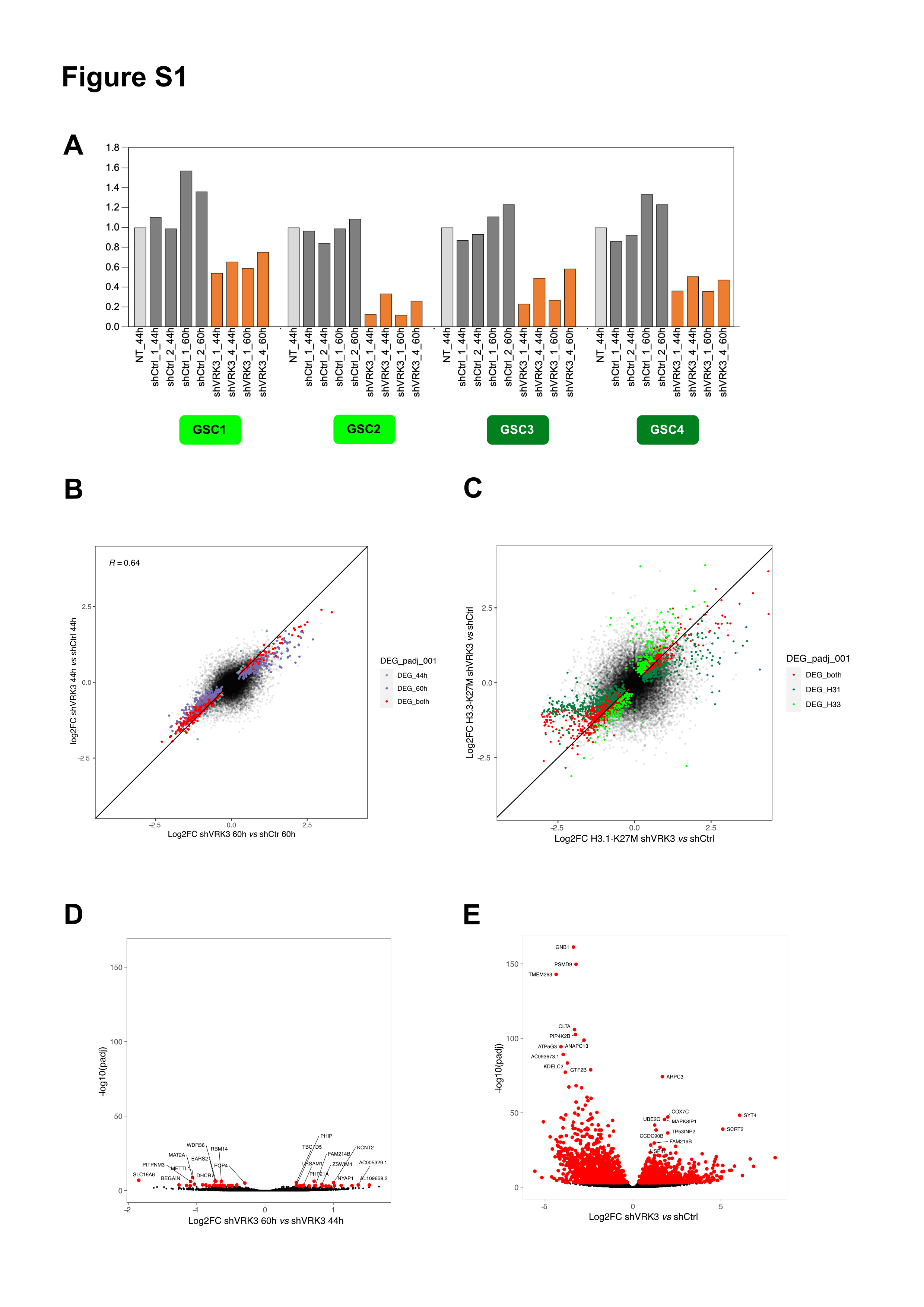

Supplement: Supplementary file 1 [file DataSheet_1.zip › Supplementary Figures/Image 1.JPEG]

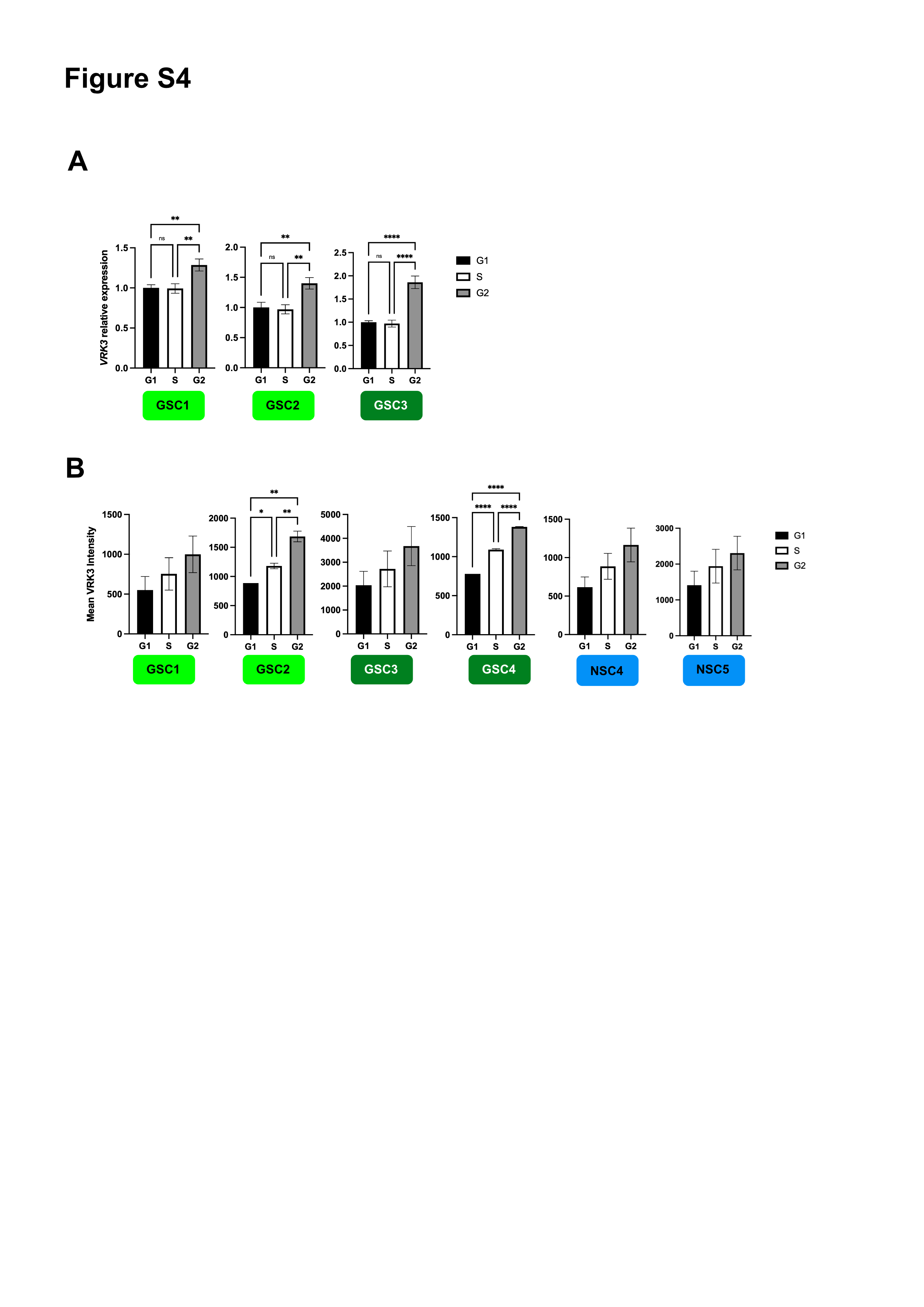

Supplement: Supplementary file 1 [file DataSheet_1.zip › Supplementary Figures/Image 4.JPEG]

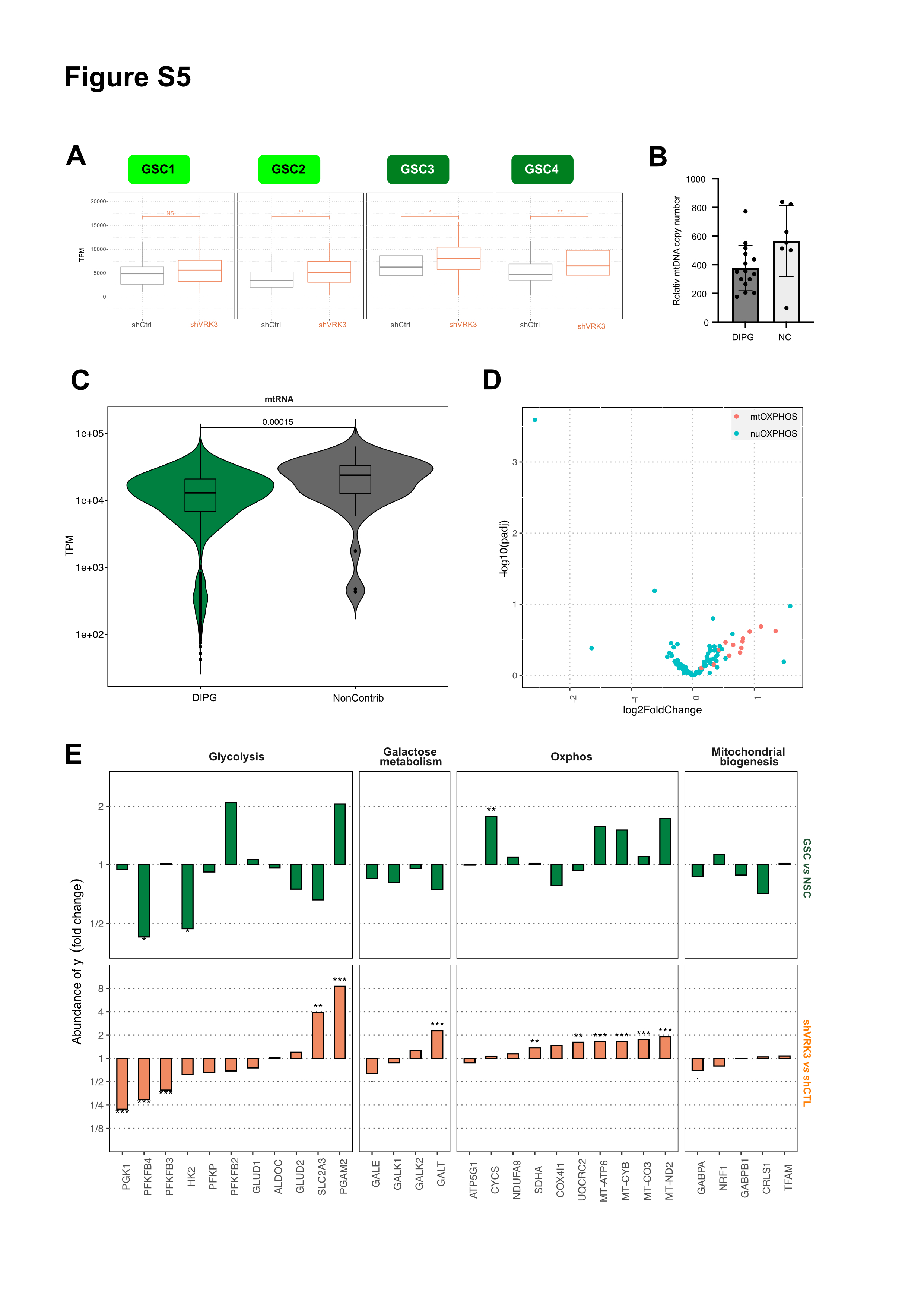

Supplement: Supplementary file 1 [file DataSheet_1.zip › Supplementary Figures/Image 5.JPEG]

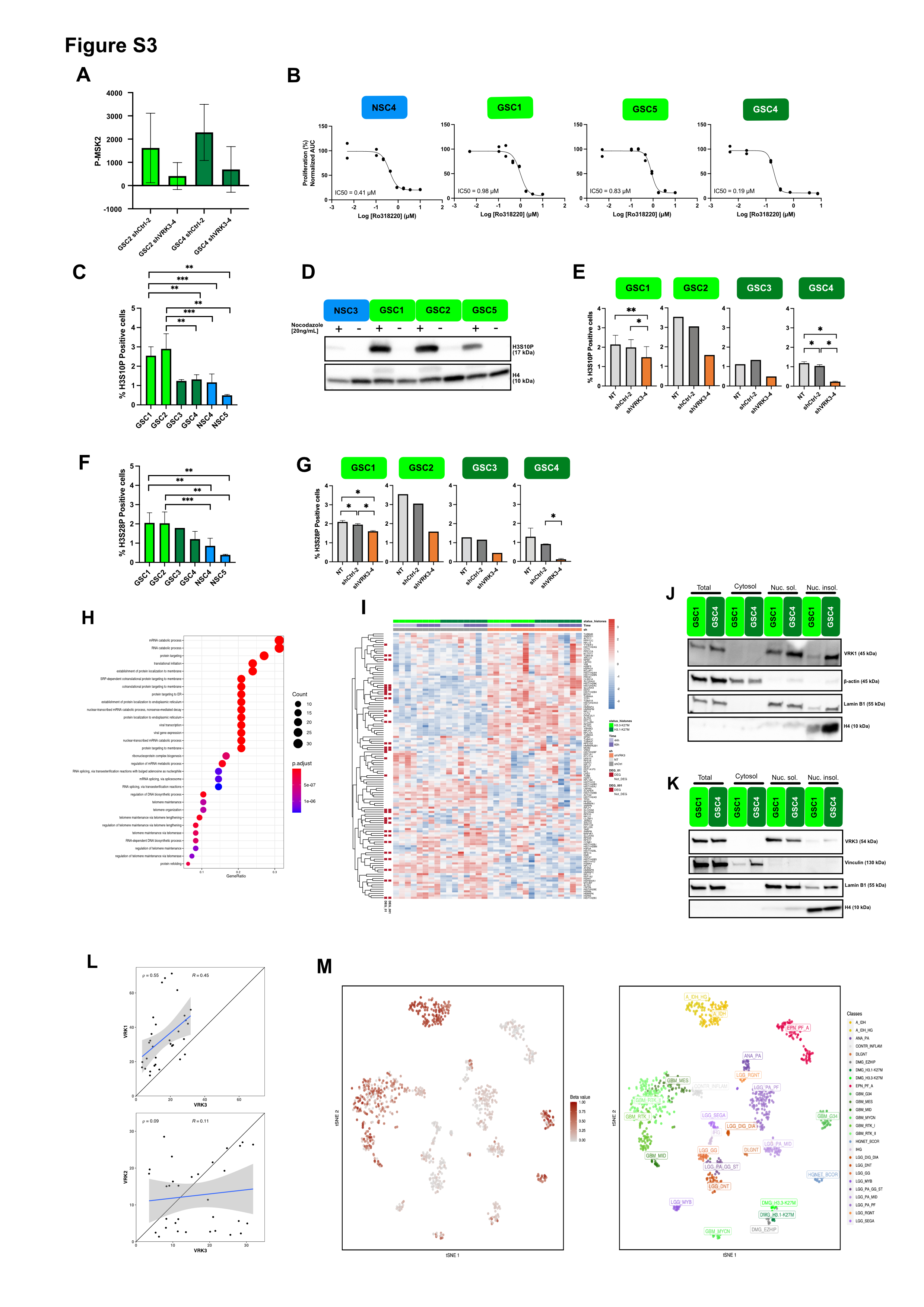

Supplement: Supplementary file 1 [file DataSheet_1.zip › Supplementary Figures/Image 3.jpg]
